# Comparative permissiveness of *Anopheles stephensi* colony mosquito with an* Anopheles arabiensis* colony to* Plasmodium falciparum* gametocytes in Metehara, Ethiopia

**DOI:** 10.1186/s13071-026-07463-5

**Published:** 2026-09-22

**Authors:** Zewdu Selemon Amare, Temesgen Ashine, Solomon Sisay, Melaku Petros Markos, Fikregabrail Aberra Kassa, Tirhas Endale, Lemesa Ejeta, Tigist Atele, Getinet Habtamu, Natnael Lemessa Hundera, Matiwos Kidane Ayele, Betelhem Akililu Kebede, Melat Abdo, Abena kochora, Muluken Assefa, Mulugeta Demisse, Abrham Gashaw, Sagni Chali, Hassen Mamo, Fekadu Massebo, Fitsum G. Tadesse, Elifaged Hailemeskel

**Affiliations:** 1 Malaria and NTD Division, Armauer Hansen Research Institute, Jimma Road, PO Box 1005, Addis Ababa, Ethiopia; 2 Arba Minch University, Arba Minch, Ethiopia; 3 Addis Ababa University, Addis Ababa, Ethiopia; 4 Radboud University Medical Center, Nijmegen, The Netherlands; 5 London School of Hygiene and Tropical Medicine, London, UK; 6 Wollo University, Dessie, Ethiopia

**Keywords:** *Anopheles stephensi*, *Anopheles arabiensis*, *Plasmodium falciparum*, Direct membrane feeding assay, Infectivity, Permissiveness

## Abstract

**Background:**

The establishment of *Anopheles stephensi* in Africa has raised concerns about its potential role in transmitting local *Plasmodium falciparum* strains relative to native primary vectors. To address this, we evaluated the permissiveness of a newly established *An. stephensi* colony in comparison with a laboratory-adapted *An. arabiensis* colony, the principal malaria vector in Ethiopia, using paired direct membrane feeding assay (DMFA).

**Methods:**

We recruited 43 patients with microscopy-confirmed *P. falciparum* gametocytes at Metehara Health Center, central Ethiopia. Fresh venous blood samples were used to assess infectivity by feeding 3–5-day-old female *An. stephensi* and *An. arabiensis* colony mosquitoes in paired DMFA; 10 days post-feeding, oocyst development was examined microscopically. Vector permissiveness was determined by the proportion of oocyst-positive mosquitoes and the intensity of midgut oocyst infection.

**Results:**

*An. arabiensis* exhibited higher feeding efficiency (median 85.0%, interquartile range [IQR] 80–89%) compared with *An. stephensi* (78.3%, IQR 73–85%; *P* = 0.008). The proportions of feeds resulting in at least one infected mosquito were comparable between *An. stephensi* (69.7%, *n* = 30) and *An. arabiensis* (67.4%, *n* = 29; *P* = 0.94). A strong correlation was observed in infection rates between the two colonies (Spearman’s *ρ* = 0.85; *P* < 0.001). Median oocyst density was similar for *An. stephensi* (13.5 per infected mosquito, IQR 3.7–44.5) and *An. arabiensis* (26.3 per infected mosquito, IQR 10.4–89.3; *P* = 0.083).

**Conclusions:**

*An. stephensi* demonstrated permissiveness comparable to *An. arabiensis* for *P. falciparum* strains from symptomatic patients in Ethiopia. With the ongoing expansion of *An. stephensi* in Africa, further investigations are needed to elucidate its role in malaria transmission within newly invaded ecological settings.

**Graphical Abstract:**

**Figure:**
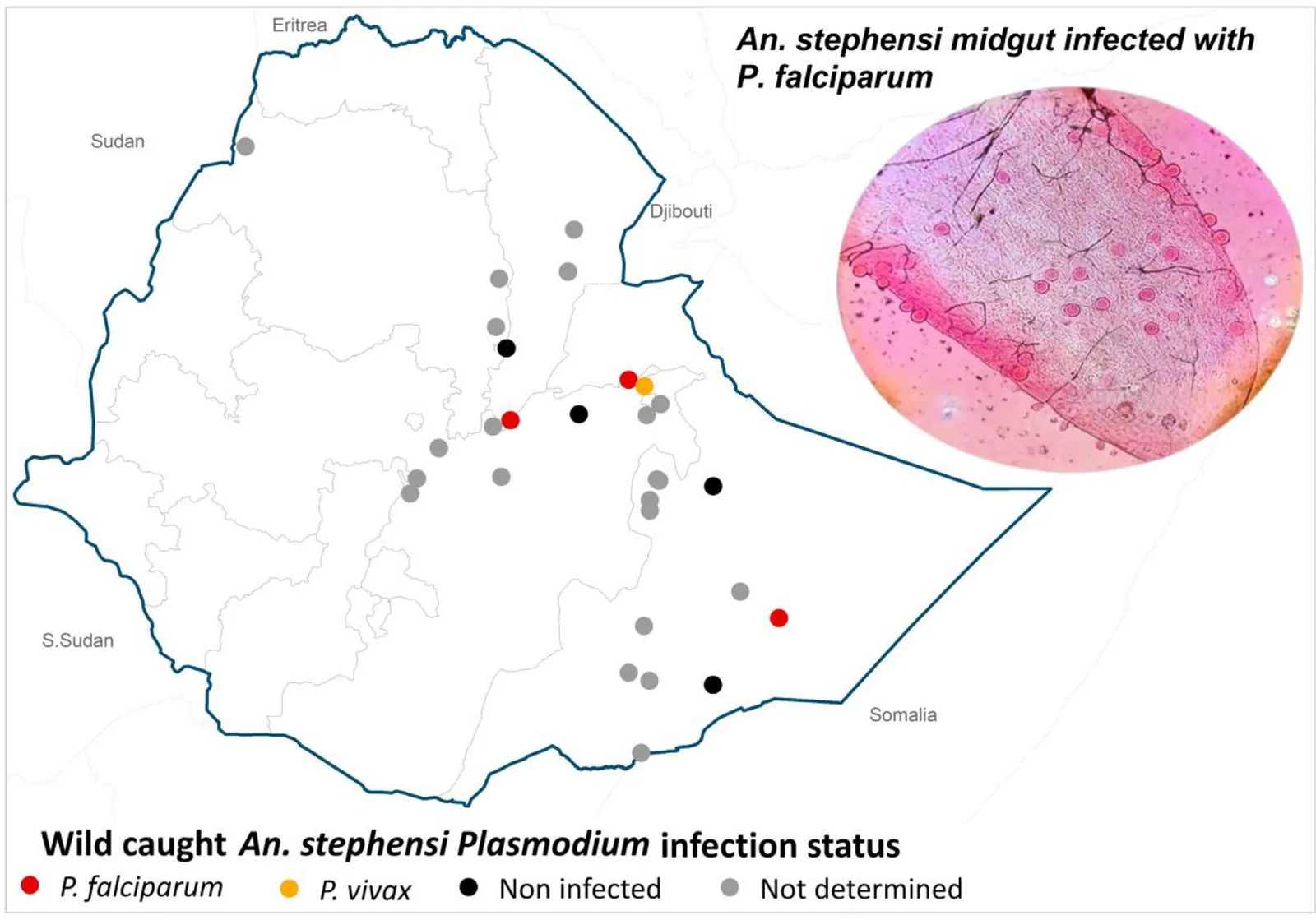

**Supplementary Information:**

The online version contains supplementary material available at https://doi.org/10.1186/s13071-026-07463-5.

## Background

Malaria remains a major public health challenge despite substantial progress achieved over the past two decades [1]. In 2024, an estimated 282 million malaria cases and 610,000 deaths were reported globally; an increase of 9 million cases compared with 2023 [2]. Sub-Saharan Africa continues to bear the overwhelming burden, accounting for approximately 94% of global malaria cases [3]. Persistent transmission is sustained by highly efficient vectors such as *Anopheles gambiae* complex and *Anopheles funestus,* further compounded by widespread insecticide resistance [4]. Control and elimination efforts are increasingly threatened by the emergence of parasites that evade detection by rapid diagnostic tests (RDTs) and exhibit resistance to first-line artemisinin-based combination therapies (ACTs) [5, 6]. Moreover, the recent invasion of *An. stephensi*, an Asian malaria vector, poses a new and significant challenge to malaria control and elimination strategies in Africa [7].

Since its first detection in 2012 in Djibouti [8], *An. stephensi* has expanded across the Horn of Africa and beyond, including Ethiopia (2016) [9], Sudan (2019) [10], Eritrea (2019) [11], Somalia (2019) [12], Nigeria (2020) [13], Yemen (2021) [14], Kenya (2022) [15], Ghana (2022) [16], and Niger (2025) [17]. In its native range across the Indian subcontinent and the Persian Gulf [8], it is a competent vector for both *Plasmodium vivax* and *Plasmodium falciparum* [13], although its efficiency varies geographically, being relatively low in regions such as Myanmar, China, and Thailand [18].

*P. falciparum* originated in Africa and adapted to diverse vectors during its global spread [19]. The recent establishment of *An. stephensi* in Africa raises critical concerns about its ability to transmit local *P. falciparum* strains. Evidence suggests that Asian *P. falciparum* strains infect *An. stephensi* more efficiently than African strains infect *An. gambiae* [20, 21], underscoring the need to assess compatibility between African parasites and this invasive vector. With the ongoing expansion of *An. stephensi* into new ecological niches in Africa and its potential link to malaria transmission [22, 23], evaluating its permissiveness to local parasites has become a priority.

Initial evidence on the susceptibility of *An. stephensi* to African malaria parasites emerged in 2021 through direct membrane feeding assays (DMFA) comparing *An. stephensi* and the native *An. arabiensis* [24]. That study primarily focused on *P. vivax*, and the limited number of *P. falciparum* observations precluded firm conclusions. Given that *P. falciparum* and *P. vivax* are co-endemic in the Horn of Africa, contributing approximately 60% and 40% of clinical malaria cases in Ethiopia, respectively [25], further investigation into the role of *An. stephensi* in transmitting local *P. falciparum* strains is warranted, particularly in light of reports linking its introduction to malaria resurgence in Djibouti and Ethiopia [22, 23].

The primary objective of this study was to compare the permissiveness of a newly established *An. stephensi* colony with a well-established *An. arabiensis* colony in Ethiopia using DMFA on blood from symptomatic patients carrying *P. falciparum* gametocytes.

## Methods

### Description of study area

**Figure 1:**
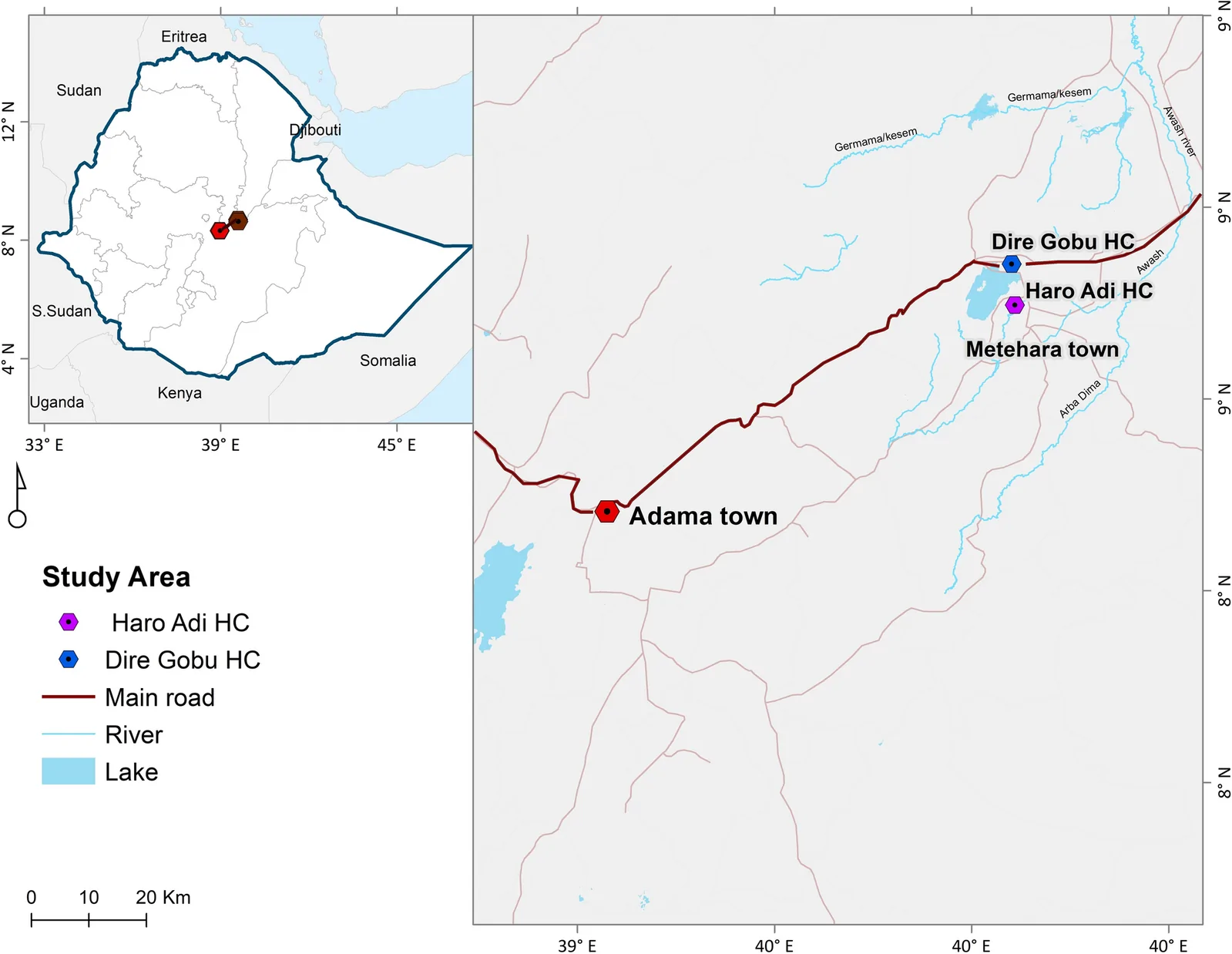
Map of the study site, Metehara town, and the insectary site (Adama)

The study was conducted in Metehara town in Oromia Regional State, central Ethiopia, from August 2024 to January 2025. Metehara lies at 08° 54′ N, 39° 55′ E, with an elevation of approximately 947 m above sea level. Malaria transmission is seasonal, with peak transmission occurring from August to December following the major rainy season from June to August [26]. The area is co-endemic for *P. vivax* and *P. falciparum*, with the latter accounting for about 75% of clinical infections [26]. The principal native vectors include *An. arabiensis, An. pharoensis*, and *An. funestus* [27]. Metehara lies along the major transportation corridor connecting Ethiopia with Djibouti. *An. stephensi* has been reported in the area since 2019 [28, 29]. Patients were recruited at two health centers in Metehara (Dire Gobu and Haro Adi) (Fig. 1).

### Mosquito rearing at Adama Malaria Research and Training Center

The Adama Malaria Research and Training Center has maintained *An. arabiensis* colonies since the 1970s. To support studies on the biology of the invasive vector, *An. stephensi* colony was established at Adama insectary in April 2023 and had reached the twenty-fifth generation at the start of the DMFA. Both *An. arabiensis* and *An. stephensi* colonies were maintained as described before [30]. Briefly, adult mosquitoes were maintained on sterile 10% sugar solution under controlled temperature, 25 ± 2°C, and relative humidity (RH), 70 ± 10%. For DMFA, 3–5-day-old mosquitoes from both colonies were transported to Metehara in insect rearing cages (BugDorm-4 Insect Rearing Cage; 24.5 × 24.5 × 24.5 cm). During transport, cages were secured inside a custom-made rectangular metal frame and covered with a wet towel to maintain temperature and humidity. This setup ensured stable conditions for mosquito survival during transportation and throughout their maintenance in Metehara before and after feeding.

### Direct membrane feeding assay at Metehara

Patients presenting at the two health facilities with uncomplicated malaria who were microscopy positive for *P. falciparum* gametocytes (at least 16 gametocytes/µL) were invited to participate in the study after providing written informed consent. Each participant donated a 5 mL venous blood sample prior to receiving antimalarial treatment (artemether-lumefantrine) [31]. Of this, 2 mL was collected in Lithium Heparin (BD Vacutainer®) tubes and immediately used for DMFA, while the remaining 3 mL was collected in ethylenediaminetetraacetic acid (EDTA)-coated tubes and preserved for parasitological and molecular analyses.

Age-matched female *An. stephensi* and *An. arabiensis* mosquitoes (3–5 days old) were fed in parallel on the same patient blood after being starved for 6–8 h. For each experiment, 120 mosquitoes per species were used (40 per cup in 3 cups). Feeding was conducted in the dark for 25–30 min using water-jacketed glass feeders (mini-feeder, Coelen Glastechniek, the Netherlands) covered with artificial membranes (Parafilm) and connected to a circulating water bath (Julabo) maintained at 37 °C. Fully engorged mosquitoes were transferred to post-feeding maintenance cages (custom made, 20 × 20 × 20 cm), which were placed under metal frames covered with wet towels. Mosquitoes were held under these conditions in Metehara for 24 h prior to transportation to Adama Malaria Research and Training Center insectary.

On day 10 post-feeding, 30 randomly selected mosquitoes from each species were dissected to examine oocysts, following established protocols [32, 33]. When fewer than 30 mosquitoes survived, all were dissected. Remaining mosquitoes were maintained until day 14 post-feeding and preserved for sporozoite detection, with analysis focused on experiments showing low oocyst prevalence (< 20%). Dissected midguts were stained with 1% mercurochrome solution prepared in phosphate-buffered saline solution, and oocysts were counted under a stereomicroscope at 40× magnification.

### Quantification of *Plasmodium* species

Asexual parasites were counted against 200 white blood cells (WBCs), and gametocytes against 500 WBCs by a World Health Organization (WHO)-certified malaria microscopist. Parasite and gametocyte densities were then calculated per microliter of blood using an assumed white blood cell (WBC) count of 8000/µL.

Genomic DNA was extracted from dried blood spot (DBS) using a QIAGEN kit (QIAGEN, Germany) to confirm and quantify *Plasmodium* infection by targeting the small 18S ribosomal subunit gene of *P. falciparum* and *P. vivax* in a multiplex real time quantitative polymerase chain reaction (qPCR) [34]. For gametocyte quantification, RNA was extracted from 50 µL cell pellet preserved in 250 µL RNA protect using RNeasy® Kit (QIAGEN, Germany) following the manufacturer’s protocol [35]. The RNA eluate was used in a multiplex reverse transcriptase qPCR assay targeting the *PfMGET* (PF3D7_1469900) marker for male gametocytes and *CCP4* (PF3D7_0903800) marker for female gametocytes [36].

To supplement microscopy-based oocyst detection at low oocyst densities, where visual inspection can occasionally be affected by artifacts [37], and to confirm the potential of sporozoite development from low-density infections (< 20% infection prevalence; 12 experiments with *An. stephensi* and 10 with *An. arabiensis*), mosquito carcasses preserved on day 14 post-feeding were homogenized using a bead-beater with 0.5 mm silica beads [38]. DNA was extracted using cetyltrimethylammonium bromide (CTAB) [39]. *Plasmodium* infections were confirmed by 18S qPCR, as described above.

### Statistical analysis

Data were analyzed using Stata version 17 (StataCorp LLC) and R version 4.4.3 (R Foundation for Statistical Computing). Descriptive statistics included proportions, geometric means (GM) with 95% confidence intervals (CIs), and medians with interquartile ranges (IQRs). Feeding efficiency (proportion of fully engorged mosquitoes) and oocyst burdens were compared between *An. stephensi* and *An. arabiensis* using Wilcoxon rank-sum tests. The Spearman’s correlation assessed relationships between mean oocyst density and infection prevalence for each mosquito species. The Pitman–Morgan test evaluated equality of variances in malaria infection rates between the two species. All tests employed two-tailed analyses with *α* = 0.05.

## Results

### Characteristics of study participants

A total of 43 microscopically confirmed *P. falciparum* gametocyte positive patients were recruited for the study. Among these, 41 patients were *P. falciparum* mono-species infected and the remaining (*n* = 2) were mixed-species infected (*P. falciparum* and *P. vivax*) (Table 1). Further confirmation by 18S qPCR showed that 40 infections were *P. falciparum* and 3 were mixed-species infections. The median age of the participants was 16 years (IQR 9–31 years) and 51.1% were male.

**Table 1:** Characteristics of study participants

Attribute	Diagnosis	*P. falciparum*
Number of positive patients	Microscopy	43*
	18S qPCR	43*
Sex, % male (*n*)	–	51.1 (22)
Age (years), median (IQR)		16.0 (8.0–31.0)
Asexual parasites density/μL, GM (95% CI)	Microscopy	5747.5 (3051.5–10,825.4)
	18S qPCR	3225.6 (2011.3–5172.9)
Gametocyte density/μL, GM (95% CI)	Microscopy	632.6 (405.1–987.7)
Male gametocyte density/μL, GM (95% CI)	RTqPCR	44.0 (95% CI 21.9–88.3)
Female gametocyte density/μL, GM (95% CI)	RTqPCR	50.2 (95% CI 24.61–02.5)
Gametocyte sex ratio, GM (95% CI)	RT qPCR	0.97 (95% CI 0.60–1.6)

*Two participants were mixed infection patients while three were confirmed as mixed infections by PCR. GM 95% CI, geometric mean with 95% confidence interval

All participants had *P. falciparum* gametocytes detected by microscopy (geometric mean [GM] 632.6 gametocytes/µL, 95% CI 405.1–987.7). Total parasite density by 18S qPCR (GM 3225.6 parasites/µL, 95% CI 2011.3–5172.9) was highly correlated with asexual parasite density determined by microscopy (GM 5747.5 parasites/μL, 95% CI 3051.5–10,825.4; *ρ* = 0.84, *P* = 0.001). Among participants with *P. falciparum* mono-infection, the density of female gametocytes was higher (GM 50.2, 95% CI 24.6–102.5) compared with male gametocytes (GM 44.0, 95% CI 21.9–88.3) and the male to female gametocyte sex ratio was 0.97 (95% CI 0.60–1.6; Table 1).

### Comparison of feeding efficiency and proportion of midgut infections

**Figure 2:**
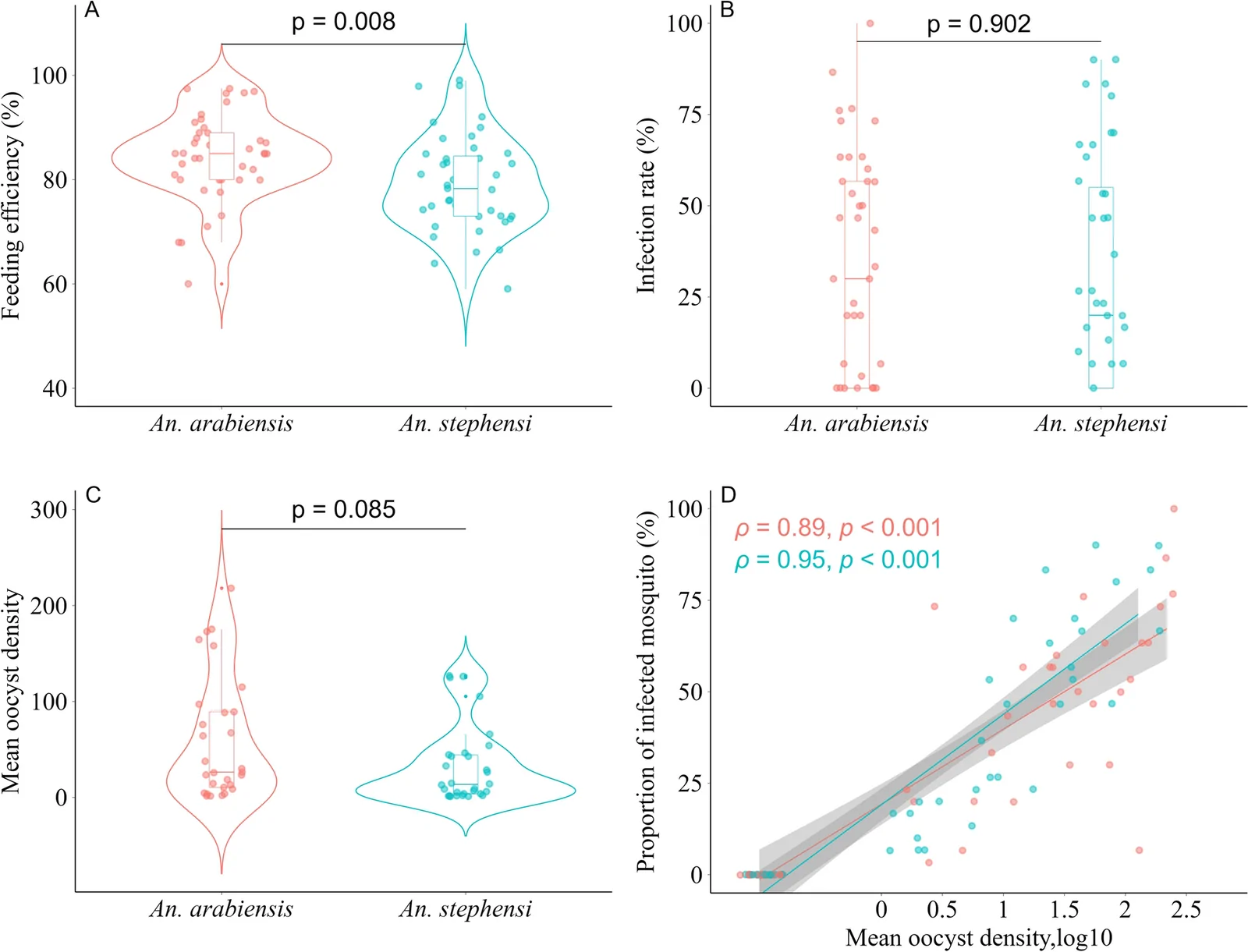
Comparison of feeding efficiency and infection proportions for *An. stephensi* and *An. arabiensis* mosquitoes in paired feedings.** A** Feeding efficiency (proportion of fully engorged) is shown together with **B** the proportion of infected mosquitoes and the mean oocyst density **C** in infected *An. arabiensis* (red) and *An. stephensi* (blue) mosquitoes. Depicted in **D** is correlation between mean oocyst density (log_10_) and proportion of infected mosquitoes for *An. stephensi* (blue) and *An. arabiensis* (red)

The proportion of feeds that infected at least one mosquito was similar between *An. stephensi* and *An. arabiensis* (67.4%, 29/43, *P* = 0.94) (Table S1). In total, 10,080 female mosquitoes (*An. stephensi,* *n* = 5040; *An. arabiensis,* *n* = 5040) with a median age of 4 days (IQR 3–5 days) were exposed to *P. falciparum* patient blood in 43 paired DMFAs. Of these, 4016 *An. stephensi* (median 94 per feeding, IQR 88–101) and 4251 *An. arabiensis* (median 100 per feeding, IQR 94–105) were fully fed. Feeding efficiency was significantly higher in *An. arabiensis* (85.0%, IQR 80–89%; *P* = 0.008) compared with *An. stephensi* (78.3%, IQR 73–85; Fig. 2A).

**Figure 3:**
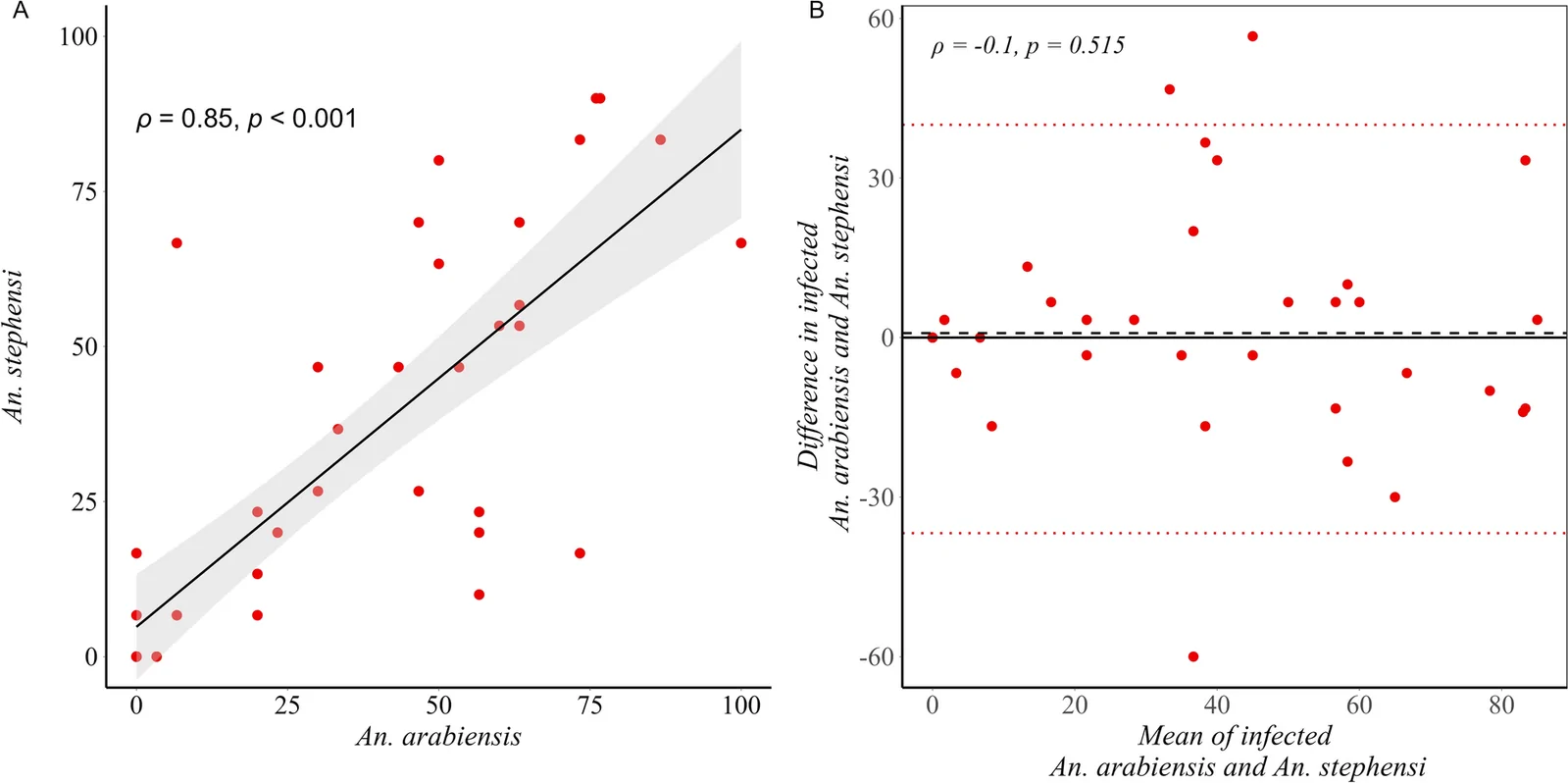
Comparison of infection rates for *An. stephensi* and *An. arabiensis* mosquitoes in paired feeding experiments in Metehara, Ethiopia.** A** Association of the percentage of infected mosquitoes for both *An. arabiensis* and *An. stephensi* from paired feeds. **B** Bland–Altman plot displaying the mean infection rate for each paired sample against the difference between the two species (*An. arabiensis* minus *An. stephensi*). The broken horizontal black line represents the mean difference infection rate (+0.84). The broken horizontal red lines show the 95% limits of agreement (−36.3 to 38.0)

Of the total mosquitoes that survived until day 10 post-feeding, 3286 *An. stephensi* and 2642 *An. arabiensis*, a total of 1290 *An. stephensi* and 1259 *An. arabiensis* were dissected, averaging 30 mosquitoes per experiment. The proportion of infected mosquitoes was identical in both species (30.7%; *An. stephensi,* *n* = 396 and *An. arabiensis,* *n* = 387). There was a strong correlation between infection rates in the two species (*ρ* = 0.85; *P* < 0.001; Fig. 3D). Median oocyst density per infected mosquito was comparable between species: *An. stephensi* (13.5, IQR 3.7–44.5) and *An. arabiensis* mosquitoes (26.3, IQR 10.4–89.3; *P* = 0.083).

### *Plasmodium falciparum* establishment and oocyst intensity among *An. stephensi *and *An. arabiensis*

A total of 19,914 oocysts were counted in 396 infected *An. stephensi* and 28,478 oocysts in 387 infected *An. arabiensis* midguts. The mean oocyst density was correlated with the proportion of infected *An. arabiensis* (*ρ* = 0.89; *P* < 0.001) and *An. stephensi* mosquitoes (*ρ* = 0.95; *P* < 0.001; Fig. 2D). Infectivity of patient blood to mosquitoes in DMFAs showed no significant bias toward either the *An. stephensi* or the *An. arabiensis* mosquito colonies (average bias 0.84; 95% limits of agreement −36.3 to 38.0; *P* = 0.515; Fig. 3B). Similarly, there was no difference in the average number of oocysts (*W* = 515; *P* = 0.083; Fig. 2C) detected in the infected midguts.

Mosquitoes from experiments with < 20% infection prevalence were randomly selected on day 14 post-feeding. In these low oocyst infection batches, 120 *An. stephensi* and 100 *An. arabiensis* mosquitoes were tested for sporozoite positivity using 18S qPCR. Comparable proportions of mosquitoes were found to be infected: 14.2% (*An. stephensi,* *n* = 17) and 10.0% (*An. arabiensis,* *n* = 10; *P* = 0.345).

## Discussion

Following the expansion of *An. stephensi* into Africa since 2012, there has been a growing interest to understand its relative role in the transmission of malaria in its new ecological range in Africa. This study compared the susceptibility of the invasive *An. stephensi* with *An. arabiensis* using paired DMFAs. Both species supported *P. falciparum* transmission at similar levels, with comparable infection prevalence, oocyst densities, and sporozoite positivity. Although *An. arabiensis* showed slightly higher feeding efficiency, overall permissiveness to infection was equivalent, confirming that *An. stephensi* can sustain local transmission as effectively as the native vector.

The blood feeding efficiency of *An. arabiensis* colony mosquitoes was higher than *An. stephensi* colonies. Such enhanced feeding propensity is a common adaptation in long-established laboratory colonies, even within the same mosquito species [40]. This is likely driven by inbreeding and selection for traits favoring metabolic activity and lab adaptability [41]. Consistent with this, our earlier work [30] showed that wild *An. arabiensis* were less efficient in blood feeding compared with established *An. arabiensis* colony, although infection prevalence and oocyst burden with *P. vivax* did not differ between wild and the colony mosquitoes.

In our earlier work [24], we demonstrated that *An. stephensi* is permissive to both *P. falciparum* and *P. vivax* relative to *An. arabiensis* using paired DMFAs, although limited numbers of *P. falciparum* feeds restricted direct comparisons. Because gametocyte maturation requires 9–12 days [42], recruiting *P. falciparum*-gametocyte-positive patients is challenging. In the present study, we addressed this gap by enrolling gametocyte-positive *P. falciparum* patients in Metehara, where *An. stephensi* has been established since 2019 [28, 29], enabling a robust comparison of susceptibility between the invasive and native vector colonies.

The intensity of oocyst establishment was comparable between *An. stephensi* and *An. arabiensis*. Infection proportions were nearly identical (30.7% in both species), and infection rates in paired feedings were strongly correlated (*ρ* = 0.86; *P* < 0.001), confirming equal susceptiblity to local *P. falciparum* strains under controlled conditions. These results underscore the epidemiological significance of *An. stephensi* as an emerging vector capable of sustaining transmission at levels equivalent to the native species.

We examined experiments with low oocyst prevalence (< 20%) to validate microscopy and assess whether such infections could progress to sporozoite development. Although a single oocyst can yield thousands of sporozoites, the critical question was whether infections with only a few oocysts per midgut (2–5, as often seen in natural transmission settings [43]) reliably result in sporozoite positivity. Our findings confirmed that even at these low densities, both *An. stephensi* and *An. arabiensis* were capable of producing sporozoites, demonstrating that low-level infections, where microscopy is most prone to false-positives, can nonetheless complete development to the sporozoite stage.

The findings of this study should be interpreted in light of certain constraints. Feeding assays were conducted under controlled conditions (25 ± 2°C; 70 ± 10% relative humidity), which do not fully reflect the environmental variability encountered by mosquitoes in a natural setting. Such fluctuations can influence physiology and oocyst establishment, potentially altering infection outcomes [44]. In addition, all blood donors were symptomatic individuals with relatively higher parasite densities, whereas in sub-Saharan Africa, the majority of onward *P. falciparum* transmission arises from asymptomatic reservoirs [45]. Because asymptomatic infections often occur at lower parasite densities, future work should examine whether mosquito permissiveness under these more representative transmission conditions.

## Conclusions

Taken together, our study demonstrated that *An. stephensi* is permissive as the primary vector, *An. arabiensis*, to local *P. falciparum* strains from symptomatic patients in Ethiopia. With its recent expansion in urban settings across Africa, these findings underscore the urgent need for early surveillance and targeted control strategies to curb further spread of this invasive vector.

## Supplementary Information

Additional file1

## Data Availability

The data supporting the conclusions of this study are available using this link https://github.com/AHRI-Malaria/stephpf.
