# Supplementary material for: Comparative permissiveness of Anopheles stephensi colony mosquito with an Anopheles arabiensis colony to Plasmodium falciparum gametocytes in Metehara, Ethiopia
Source: Parasit Vectors. 2026 Sep 22;19:403. doi: 10.1186/s13071-026-07463-5 (PMC13599085; doi:10.1186/s13071-026-07463-5)
Supplement: Supplementary file 1 [file 13071_2026_7463_MOESM1_ESM.docx]

**Table S1:** The feeding efficiency and oocyst infection prevalence and intensity of *An. stephensi* and *An. arabiensis* in matched membrane feeding experiment, Metehara, Ethiopia 2024.

| Attributes | *An. stephensi* | *An. arabiensis* | *p-value* |
| --- | --- | --- | --- |
| Exposed mosquitoes for feeding | 5,040 | 5,040 | - |
| Feeding efficiency, % (n/N) | 80.0 (4,016/5,040) | 84.5 (4,251/5,040) | <0.0001 |
| Proportion infectious individuals, % (n/N) | 69.7(30/43) | 67.4 (29/43) | 0.843 |
| Mosquito infection rate, % (n/N) | 30.6 (396/1290) | 30.7 (387/1259) | 1.00 |
| Mean Oocyst density/infected mosquito, Median (IQR) | 13.5 (3.8-44.5) | 26.3 (10.4-89.3) | 0.373 |
